# A heart shaped coronary aneurysm

**DOI:** 10.1007/s12471-025-01980-1

**Published:** 2025-08-28

**Authors:** Charlotte Snik, Mustafa Koksu-Ilhan, Saman Rasoul

**Affiliations:** Zuyderland Medisch Centrum Heerlen, Heerlen, The Netherlands

A 72-year-old woman with a history of hypertension and recently diagnosed heart failure underwent invasive coronary angiography, revealing a significant lesion of the left anterior descending with a heart-shaped aneurysm (Fig. 1). Following multidisciplinary Heart Team discussion, she underwent a successful percutaneous coronary intervention.

**Fig. 1:**
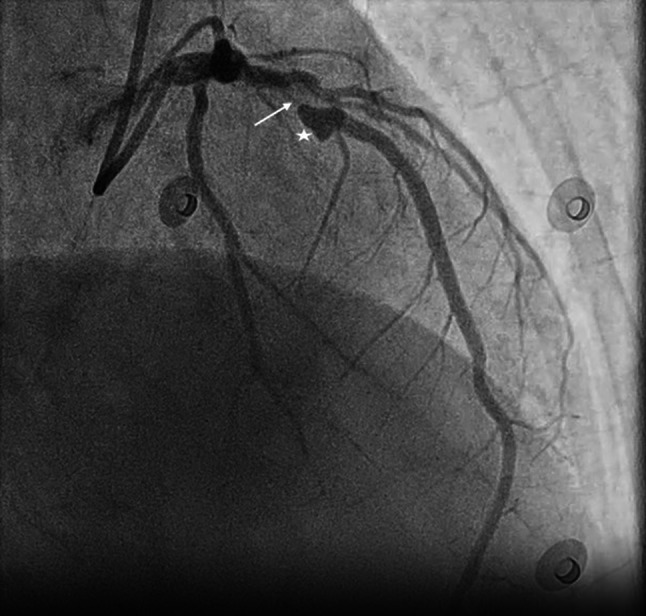
Heart-shaped coronary artery aneurysm indicated by a *star*, left anterior descending indicated by an *arrow*

Coronary artery aneurysm (CAA), a focal dilation of coronary segments of at least 1.5 times the adjacent normal segment, is found in up to 5% of patients undergoing coronary angiography [1] and is associated with poor long-term outcome [2]. CAA is usually found incidentally on cardiac imaging; however, it may present with stable angina or acute coronary syndrome [3]. Two forms exist: saccular and fusiform aneurysms. The pathogenesis is not well known. Genetic factors, atherosclerotic risk factors, certain vasculitides, and iatrogenic factors may be the cause [3]. Management is challenging and includes medical therapy, surgical excision, coiling, percutaneous coronary intervention, and coronary bypass grafting [4].

## Supplementary Information

Figure series from the case studies.
